# Off-label use of clomiphene citrate to treat anabolic androgenic steroid induced hypogonadism upon cessation among men (CloTASH) – A pilot study protocol

**DOI:** 10.1016/j.mex.2024.102810

**Published:** 2024-06-19

**Authors:** Ingrid Amalia Havnes, Hans Christian Bordado Henriksen, Per Wiik Johansen, Astrid Bjørnebekk, Sudan Prasad Neupane, Jonny Hisdal, Ingebjørg Seljeflot, Christine Wisløff, Marie Lindvik Jørstad, Jim McVeigh, Anders Palmstrøm Jørgensen

**Affiliations:** a Division of Mental Health and Addiction, Oslo University Hospital, PO box 4959 Nydalen, Oslo 0424, Norway; b Institute of Clinical Medicine, Faculty of Medicine, University of Oslo, PO box 1171 Blindern, Oslo 0318, Norway; c Anabolic Androgenic Steroid Research Group, Section for Clinical Addiction Research, Division of Mental Health and Addiction, Oslo University Hospital, PO box 4959 Nydalen, Oslo 0424, Norway; d National Advisory Unit on Substance Use Disorder Treatment, Division of Mental Health and Addiction, Oslo University Hospital, PO box 4959 Nydalen, Oslo 0424, Norway; e National Centre for Suicide Research and Prevention, Institute of Clinical Medicine, University of Oslo, Sognsvannsveien 21, Building 12, Oslo 0372, Norway; f Oral Health Centre of Expertise in Rogaland, Torgveien 21 B, Stavanger 4016, Norway; g Department of Vascular Surgery, Oslo University Hospital - Aker, Postboks 4959 Nydalen, Oslo 0424, Norway; h Center for Clinical Heart Research, Department of Cardiology, Oslo University Hospital - Ullevål, PO box 4954 Nydalen, Oslo 0424, Norway; i Substance Use and Associated Behaviours Group, Manchester Metropolitan University, Geoffrey Manton Building, 4 Rosamond Street West, Manchester M15 6LL, United Kingdom; j Section of Specialized Endocrinology, Department of Endocrinology, Oslo University Hospital - Rikshospitalet, PO box 4950 Nydalen, Oslo 0424, Norway

**Keywords:** Anabolic androgenic steroids, Performance and image enhancing drugs, Hypogonadism, Post cycle therapy, Clomiphene citrate, Testosterone, Human chorionic gonadotropin, Intervention study, Pilot study, Proof-of-concept study, Proof-of-concept off-label hormone intervention

## Abstract

**Background:**

Non-prescribed anabolic androgenic steroid (AAS) use is associated with AAS-induced hypogonadism (ASIH), and metabolic, cardiovascular, and mental health risks. Symptoms of ASIH (fatigue, depression, anxiety, sexual dysfunction) are hard to endure following cessation, but there is no consensus on whether endocrine treatment should be used to treat ASIH. This proof-of-concept study aims to explore safety of off-label clomiphene citrate therapy, whether the treatment will reduce the symptoms of androgen deficiency, and to study changes in health risks after cessation.

**Methods:**

In this open-labeled non-randomized off-label hormone intervention pilot study, we shall include males with AAS dependence intending to cease use. The 16-week intervention included clomiphene citrate, transdermal testosterone gel for the first four weeks and optional human chorionic gonadotropin (hCG) from week 4 if low treatment response. Measures of physical and mental health will be examined from ongoing AAS use, during the intervention, and at 6- and 12 months post cessation. Change in self-reported symptoms of hypogonadism and other withdrawal symptoms will be compared with data from a group of men who ended AAS use temporarily without the medical intervention. The study may provide valuable clinical insights and may be used to inform the design of future intervention studies.

## Graphical abstract

**Figure:**
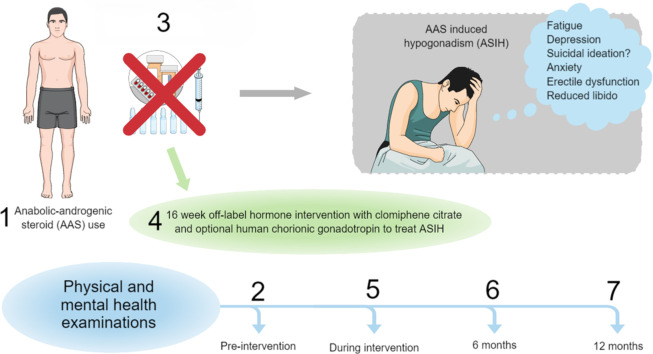

Specifications table

**Table:** 

Subject area:	Medicine and Dentistry
More specific subject area:	Endocrinology, anabolic androgenic steroid induced hypogonadism
Name of your protocol:	Off-label use of Clomiphene citrate to Treat Anabolic-androgenic Steroid induced Hypogonadism upon cessation among men (CloTASH) - a pilot study protocol
Reagents/tools:	Clomiphene citrate, testosterone, human chorionic gonadotropin
Experimental design:	Open label non-randomized off-label hormone intervention study
Trial registration:	EudraCT 2020–005938–15
Ethics:	The research will be carried out according to the Helsinki declaration. The study is approved by the Regional Committee for Medical and Health Research Ethics (REC) in Norway (33872), Norwegian Medicines Agency (21/18081–9) and the Data Protection Officer for Research at Oslo University Hospital (20/27593). All participants will receive oral and written information about the study and written formal consent will be collected.
Value of the Protocol:	• This is the first study to test feasibility of off-label use of clomiphene citrate with the intention to restart endogenous testosterone production upon cessation of anabolic androgenic steroids (AAS) among men with AAS induced hypogonadism. • The study may provide valuable clinical insights, enabling the exploration of whether adjustments are needed for the intervention. • The results may be used to determine the sample size and informing the design of future RCTs or case comparison studies.

## Background

Non-prescribed AAS are predominantly utilized by male recreational athletes in cyclic patterns, with intermittent breaks or continuous administration. Global lifetime prevalence of AAS use among men is estimated to be 6.4 %, but is found higher in subpopulations [1] and associated health risks and behavioral outcomes make AAS use a public health concern [2]. The wide range of adverse effects on mental [3] and physical health [[4], [5], [6]] includes negative effects on the cardiovascular system such as ventricular dysfunction [7], myocardial hypertrophy [8], hypertension [9], severe biventricular cardiomyopathy [10], acute myocardial infarction and sudden cardiac death [8,11]. Increased carotid intima-media thickness, reduced arterial elasticity and lower carotid artery compliance are early predictors of atherosclerosis and cardiovascular risk that are found among men with long term AAS use [12]. AAS use is also associated with altered renal, hepatic and metabolic functions [13]. High androgen levels might lead to gynecomastia and several dermatological conditions, and infertility during ongoing use is common due to the disruption of hypothalamic-pituitary-gonadal (HPG) axis [14,15].

One in three people using AAS seem to develop a dependence syndrome characterized by a pattern of escalating doses and reduced breaks between cycles, or continuous use despite experiencing adverse effects and a desire to cease use [16]. The neuroendocrine mechanism of AAS dependence is linked to suppression of the hypothalamic-pituitary-gonadal (HPG) axis leading to AAS-induced hypogonadism (ASIH) [14]. The features of androgen deficiency (fatigue, depression, anxiety, and sexual dysfunction) will first occur weeks after AAS cessation. The endogenous testosterone production typically recovers after 3–12 months, but this phase is hard to endure [17], often resulting in restart of AAS use.

Despite experiencing health problems associated with AAS use, many individuals fail to receive treatment [18,19]. This reluctance to engage with health services may be attributed to limited treatment options and insufficient awareness of AAS among clinicians. Many individuals who use AAS feel stigmatized, and fear being identified with or labelled as either drug users or sport cheats, with this acting as a further barrier to service engagement [20]. Instead of seeking health services, people who use AAS commonly seek knowledge among peers including in online communities. When AAS use is ceased, some may self-initiate Post Cycle Therapy (PCT) with non-prescribed hormonal substances in various combinations and doses with the intention to restore the HPG-axis faster. As one of few countries, Norway has integrated AAS and other performance and image enhancing drugs (PIEDs) in the National Drug Policy. Possession and use of AAS and other PIEDs became criminalized in 2013, and people with current or previous AAS use got access to SUD treatment in the specialist health service. There is no consensus, nationally or internationally, on whether endocrine treatment should be used in the treatment of ASIH, but endocrinologists have proposed potential interventions [21]. There is a knowledge gap regarding treatment of this patient group, and we describe a non-randomized proof-of-concept study to test a 16 weeks off-label hormone intervention for men who struggle to cease AAS use.

## Description of protocol

### Aims

The primary aim of the present study is to explore whether the 16 weeks therapy model with clomiphene citrate (CC) leads to stimulation of endogenous luteinizing hormone (LH) and testosterone production, if the treatment model is safe, and if the model is more effective in reducing AAS withdrawal symptoms compared to no intervention. The secondary aims are to detect health risks during ongoing AAS use in the intervention group only and assess whether and to what extent the health risks are reduced 12 months after cessation of AAS.

## Methods and analysis

In this open-labeled non-randomized proof-of-concept intervention study, we aim to include 25–30 AAS-dependent men who struggle to cease their AAS use. The subjects in the intervention group will receive 25 mg chlomiphene citrate every second day following AAS cessation for 16 weeks. The comparison group comprise participants in another study of men who ended AAS use temporarily without the intervention and the groups will be compared on self-reported withdrawal symptoms.

### Participants

Inclusion criteria for the intervention group comprise men above 18 years of age with continuous AAS use, AAS dependence, a desire to cease use and previous unsuccessful attempts to cease use. Men with cardiovascular conditions, severe mental health problems or severely reduced liver function will be excluded from the off-label hormone intervention. Men above 18 years of age who use AAS in cycles with breaks in between may be included in the comparison group. For more details about inclusion and exclusion criteria for the intervention group and comparison group, see Table 1.

**Table 1:** Inclusion and exclusion criteria for the intervention group and the comparison group.

	**Intervention group**	**Comparison group**
**Inclusion criteria**		
Age	≥18 years	≥18 years
AAS use	Continuous AAS use ≥6months	Cycle, ≥6weeks between cycles
Dose	Supraphysiological	≥400 mg/week
AAS dependence	3 or more items last 12 months: tolerance, withdrawal, increased amount, unsuccessful efforts to reduce or cease use, ↑time to obtain/use substances, ↓social/recreational/occupational activities due to use, continued AAS use despite mental and/or physical side effects	No
Plan to cease AAS use	Wish to cease use, previous unsuccessful attempts	No
SUD treatment	Being enrolled in or referred to Substance Use Disorder (SUD) treatment	No
Hormonal and liver status at intervention start	Serum testosterone < 25 nmol/l, liver enzymes alanine transaminase (ALT) and aspartate transaminase (AST) < 3 x upper limit of normal range (ULN) (normal range ALT 10–70 U/L, AST 15–45 U/L)	No
**Exclusion criteria**		
Mental health	Previous or current: Severe depression, bipolar disorder, psychosis	Reduced cognitive function (IQ<80) and/or previous or current: Severe depression, bipolar disorder, psychosis. Use of medications that may impact CNS functioning (e.g., antipsychotics, methyl phenidate, TRT)
Physical health	Hypersensitivity to clomiphene citrate, hyperprolactinemia, untreated thyroid or adrenal disease, hemoglobin >18.0 g/dl, current/previous thromboembolic disease, cardiovascular disease (arrhythmia, ischemic heart conditions, heart failure)	Traumatic brain injury with consciousness >1 min, or neurological disorders (e.g., multiple sclerosis, ischemia) or severe somatic disorders (e.g. cancer)
Substance use	No illicit substance use during intervention, <8 alcohol units/week	Problematic use of alcohol or illegal substances (evaluated with Alcohol Use Disorders Identification Test and Drug Use Disorders Identification Test C)

Recruitment for the intervention group**:** The National Steroid Project at Oslo University Hospital (OUH) will recruit participants for the intervention study through a national information service [24], the outpatient SUD clinic at OUH, flyers in various gyms and through advertisements on social media; Facebook, Instagram, TikTok, Snapchat and AAS user forum.

Recruitment for the comparison group**:** Advertisements on social media.

### Study design

The study includes two sub-studies with

A) *Within-subjects repeated measures design* to monitor the intervention group with physical and mental health parameters and blood sampling prior to intervention start and while still using AAS, during 16 weeks of intervention, and at follow-up at 6 and 12 months (Table 2).

**Table 2:** Flow chart of study events during the study period for the intervention group, modified version of SPIRIT.

**Fig. 1:**
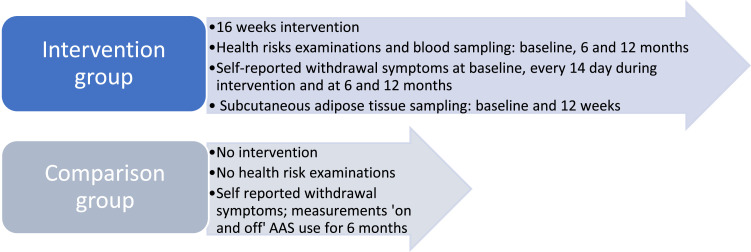
Timeline for data collection for the intervention group and the comparison group.

B) *Single center – single group – case-comparison design*:

Male participants with AAS dependence and continuous AAS use with the intention to cease use permanently comprise the intervention group. The data from the intervention group will be compared with data from an ongoing study of men who intend to cease a cycle of AAS use temporarily without receiving intervention. Participants from both studies will self-report withdrawal symptoms and other subjective health measures during ongoing use and after cessation. The intervention group will self-report withdrawal symptoms every 2 weeks for 16 weeks of intervention, see Fig. 1.

### The intervention

Rahnema and colleagues presented a treatment model to prevent severe symptoms of hypogonadism during cessation of AAS including use of CC, a Selective Estrogen Receptor Modulator (SERM), to restart the HPG-axis [14]. CC selectively binds to estrogen receptors and acts as a receptor antagonist with week estrogen activity in the hypothalamus which leads to blocked negative feedback inhibition. This leads to increased gonadotropin-releasing hormone (GnRH) pulsation in the hypothalamus, increased release of luteinizing hormone (LH) from the pituitary gland and finally increased levels of endogenous testicular testosterone. CC is approved for short-term treatment of female infertility. CC as off-label treatment has been found safe, tolerable and effective to improve serum levels of testosterone, symptoms of hypogonadism and infertility in men with hypogonadism of other causes than AAS use [22,23]. In the reviewed studies, dosages of 25 mg daily for up to 12 months, 50 mg daily for up to 6 months, and 25–50 mg every second day for 1, 2 and 3 years or more were administered. The majority of these investigations reported no occurrence of major side effects. Side effects reported were: headache, dizziness, mood changes, blurred vision/visual change, breast tenderness/gynecomastia, secondary polycythemia without need for phlebotomy and a few cases of elevated liver enzymes [22,23].

**Fig. 2:**
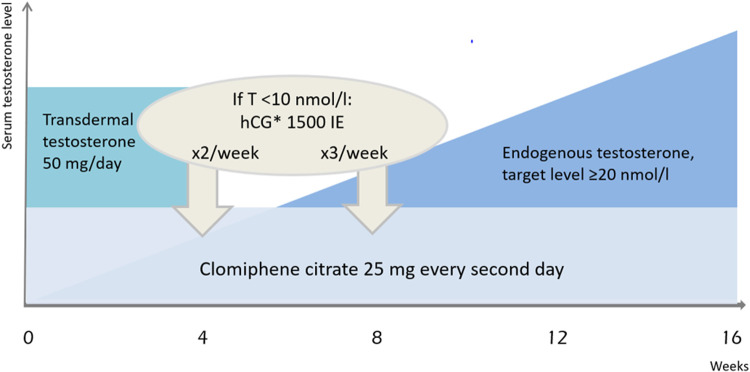
The 16 week off-label intervention with clomiphene citrate, testosterone and optional hCG *human chorionic gonadotropin.

This protocol involves a slightly modified version of the 16 week therapy model outlined by Rahnema and colleagues involves administration of 25 mg CC orally every second day for 16 weeks [11]. Testosterone 50 mg is given transdermal daily for the first 4 weeks as rescue medication before the expected endogenous Testosterone (T) response, see Fig. 2. The target T level during the intervention is set at 20 nmol/l. If the endogenous LH and T response is poor (T < 10 nmol/l) after 4 weeks, human chorionic gonadotropin (hCG) will be added as 1500 IE subcutaneous injection twice a week, as rescue medication. If the endogenous T response is still poor after 8 weeks, the same dose will be given three times a week for four more weeks or throughout the intervention. If poor response on endogenous LH and T levels below lower limit of normal range after 16 weeks intervention *and* the participant experiences severe symptoms of hypogonadism; testosterone replacement therapy (TRT) and referral to endocrinologist will be considered.

### Outcomes and instruments

Primary outcomes:

(1) *Changes in self-reported symptoms of hypogonadism,* mental health state, and symptoms associated with AAS use. The following will be assessed for both the intervention and comparison group: fatigue, depression, suicidal ideation, anxiety, wellbeing, sexual dysfunction, sleep quality, aggression, and body dysmorphia.
(2) *Side effects related to CC* will be specifically evaluated for the intervention group.

Secondary outcomes:

Change in measures of physical health from baseline to follow-up for the intervention group:

• Weight, body mass index (BMI), degree of striae, acne, androgen alopecia and gynecomastia
• Endocrine status, metabolic status, liver and kidney function (blood tests)
• Cardiovascular status: Blood pressure, electrocardiogram (ECG), vascular morphology (carotid intima-media thickness and pulse wave velocity) and -function (flow-mediated dilation and carotid artery reactivity)
• Body composition; total fat mass, visceral and subcutaneous fat tissue, total lean mass, and bone mineral density
• Testicular volume and sperm quality
• Immunological biomarkers, analyzed from biobanked blood and adipose tissue samples

Change in measures of mental health risks from baseline to follow-up after 12 months:

• Depression and anxiety levels, AAS dependence, body image concerns, aggression, AAS use, and adherence to SUD treatment

Study participants included for the intervention will be screened for eligibility at baseline and examined to assess mental health status, comorbidity, AAS history, AAS dependence and SUDs with validated instruments, see flow chart for study events (Table 1).

#### Self-reported mental health, well-being and AAS withdrawal symptoms

To investigate mental health state, wellbeing and AAS-withdrawal symptoms after AAS cessation, the following self-reporting questionnaires will be administered via a web-app every second week for 16 weeks for both groups and at follow-up at 6 and 12 months for the intervention group: Hopkins Symptom Checklist-10 (HSCL-10) to evaluate psychological distress, suicidal ideation item from Montgomery and Åsberg Rating Scale (MADRS), items from Health-related quality of life (HRQOL) to evaluate sexual function, Shortened Fatigue Questionnaire, WHO-5 Wellbeing Index and Jenkins Sleep Scale (JSS), Body Perception Questionnaire; and Visual Analog Scale (VAS) to measure aggression, cognitive function, body image and pain. Treatment satisfaction will be assessed using VAS for the intervention group only.

#### Biological assessments for the intervention group

Biological assessments will include urinalysis, semen analysis, routine biochemistry, non-routine hormone tests, markers of glucose, lipid and bone metabolism and immunological gene expression profile.

Urine will be screened for AAS and psychoactive substances at inclusion, during intervention and at follow-up. Additionally, routine blood analyses will be conducted at the time of inclusion and every 4 weeks. These regular assessments serve both safety purposes and to monitor physiological markers associated with AAS induced side effects. Throughout the intervention period and at the 6-month and 12-month follow-ups after discontinuation of AAS usage, a comprehensive set of analyses will be carried out. These include evaluation of sex hormone levels, metabolic and hematological status, glucose, insulin production and resistance, liver and kidney function, electrolytes, and immune markers.

Blood and adipose tissue are sampled in fasting condition between 8:00 and 10:00. Blood samples, including serum, EDTA and citrated plasma and RNA (Paxgene tubes) for gene expression profiling at baseline and after 6 and 12 months are prepared and stored in a biobank at −80 °C. Adipose tissue samples are taken from the gluteal region at baseline and after 12 months, immediately snapfrozen and stored in a biobank at −80 °C until gene expression profiling.

##### Physical examination

The physical examination will encompass various assessments to evaluate change in the participants’ health status from current use, during and after the intervention and includes blood pressure (BP), heart rate (HR), Electrocardiogram (ECG), weight, height, body mass index (BMI), auscultation of heart and lungs, evaluation of striae, acne, and gynecomastia. As cardiac hypertrophy is often not detected on ECG screenings among people who use AAS [25], the participants will be referred to routine echocardiography at their local hospital.

**Semen samples** will be analyzed prior to intervention and 6 and 12 months after the intervention start to evaluate change in measures of testicular function and fertility (sperm count, morphology, motility; and vitality) during and after AAS use.

**Testicular volume** will be measured by orchidometer and ultrasound at baseline and follow up at 12 months as increased volume flowing upon cessation of long term AAS use is seen as sign of improved testicular function [26].

**The vascular morphology and -function** will be evaluated by the thickness of intima media of the common carotid artery and by the carotid artery reactivity (CAR). In addition, endothelial function and pulse wave velocity will be measured at inclusion and at follow-up with established methodology at the Section of Vascular Investigation, OUH [12].

**Body composition and fat distribution, including visceral and subcutaneous adipose tissue**, will be assessed by dual energy X-ray absorptiometry (DXA-scan) to evaluate whether there is a difference in body composition (Total lean mass (kg) (TLM), Total fat mass (kg) (TFM), Body fat %, Visceral and subcutaneous adipose tissue (VAT) (g), Android fat (g), fat distribution and bone mineral (BMD) g/cm2 with T-score and Z-score in the following sites: Lumbar spine (LS) (L1-L4), Total hip, Proximal radius, Ultradistal radius, Total body during AAS use and 12 months after AAS-cessation.

##### Biomarker analyses

From the established biobank circulating inflammatory biomarkers including selected cytokines, chemokines and adhesion molecules will be analyzed. Molecular factors in focus are interleukins, tumor necrosis factor, interferon-γ, fibroblast and endothelial growth factors, vascular cell adhesion molecule 1, monocyte chemotactic factor 1 and Brain Derived Neurotrophic Factor.

Analyses of adipose tissue will encompass a comprehensive assessment, of biochemical factors relevant to cardiovascular and other health risks, including immune markers and individual markers’ genetic expression. RNA will be isolated from both the fat tissue and blood samples for further investigation of the regulation (mRNA) of proteins of interest during AAS use and 12 months after cessation of AAS. Notably, alteration in the extracellular matrix within adipose tissue has been linked to glucometabolic disturbances, such as insulin resistance/sensitivity, diabetes, and obesity, which are all relevant health concerns related to AAS use.

#### Registration of adverse events

As a safety measure, an eye examination will be conducted at an optometrist at inclusion and after the intervention. The Common Terminology Criteria for Adverse Events (CTCAE) version 5 is used to classify adverse events at follow-up examinations every 4th week during the intervention. The participants can contact the research physicians (HCBH and IAH) by phone or mail during the intervention period for any experienced side effect(s). Any event requiring medical intervention is registered (CTCAE level 2 or above). Medication-related Severe Adverse Events (SAEs) and Suspected Unexpected Severe Adverse Reactions (SUSARS) are reported to the Norwegian Medicines Agency within 7 days for fatal/life threatening events/cases and for other SUSARs, within 15 days.

### Data analysis plan

Data analyses will follow a full analysis set, with a per protocol plan. Descriptive statistics will be used when appropriate and for reported adverse events. For between-group comparisons and to control for the effect of baseline characteristics, we plan to use General Linear Mixed Models (GLMM) with a within subject repeated measures design at baseline, 6 months and 12 months. In cases where the outcomes do not meet the normality assumption, we will apply a General Alinear Mixed Models (GAMM) approach. The results of statistical analyses will be performed using 95 % CIs, and a two-sided P value of < 0.05 will be used to indicate statistical significance. General linear models may be utilized in cases where GLMM/GAMM is considered inappropriate. Two sample *t*-tests will be used for between-group comparisons of numerical variables, when we see no need to adjust for confounding variables. Numerical data not following normal distribution will be presented as medians (25th-75th percentiles), using non-parametric tests such as the Wilcoxon rank sum tests (Mann-Whitney) to compare differences between groups. Chi^2^-tests and Fischer's exact will be applied for categorical variables. Per protocol analysis will be applied in cases of missing data to detect any discrepancy that might affect the robustness of the results. All statistical calculations and analyses will be conducted using up-to-date versions of the statistical software STATA and SPSS.

### Sample size determination

In line with the proof-of-concept study design, a small sample of up to ten cases is sufficient to evaluate if the intervention works as described in theory [14]. This approach allows for the exploration of potential side effects and the subjective experiences of the participants. Every single case within the intervention group holds significant clinical relevance and is of interest.

The first AAS case control study from our research group [27,28] found significant effects between the groups for measures of psychopathology with effect sizes ex =0.27. With a power of 80 % and a wish to detect effects of moderate level (Cohen's *d* = 0.4), a total sample of 52 participants is needed.

In the current study, the recruitment target for both the intervention group and comparison group is 25–30.

### Scientific rationale for study design

The chosen design served three primary purposes (a) to assess the feasibility and acceptability of the intervention, (b) to evaluate its safety, and (c) to examine its effect on symptoms and objective measures of hypogonadism. The cohort design for the intervention group allowed for investigating potential health risks reduction following cessation of AAS use. Although a randomized controlled trial (RCT) is considered the gold standard methodology, conducting a preliminary test of the intervention before proceeding to an RCT is essential. Additionally, this approach facilitates the determination of effect sizes for use in sample size calculations for future RCTs [29]. However, it is acknowledged that the comparison group may differ from the intervention group on certain variables, as they may not share the same intention to permanently cease AAS use.

The study exclusively recruits male participants as we investigate the effect of treatment of androgen deficiency due to AAS induced male hypogonadism. The primary endpoints (symptoms of hypogonadism such as depression, anxiety, fatigue, reduced libido, and erectile dysfunction, and other symptoms related to AAS use) were developed in collaboration with a user panel comprising people with prior AAS experience and clinicians from the outpatient SUD clinic with experience of providing treatment to the patient group. These endpoints are considered reliable clinical indicators of the intended intervention effect.

### User participants’ input into design

The research questions and design for the proposed study have been discussed and developed in collaboration with a user panel of five men with former AAS use. The research questions, design, inclusion criteria and variables (background information, health risk examinations and self-report questionnaire) for the proposed study was discussed and developed in workshops with the user panel, clinicians, and researchers. The panel strongly approved testing the proposed off-label intervention as well as health risk examinations before, during and after the intervention. The user panel were also clear that if randomized control design was chosen, it would lead to drop out in the control group. Members of the panel took part in testing of the self-report questionnaire and development of ads for social media. They also suggested recruitment strategies in certain gyms, social media and through information to general practitioners and SUD treatment facilities.

### Ethics approval and consent to participate

The CloTASH off-label pilot intervention study is approved by the Norwegian Regional Committee for Medical Research Ethics (33872), the Norwegian Medicines Agency (21/18081–9) with EudraCT-number 2020–005938–15 and the Data Protection Officer at Oslo University Hospital (20/27593). All participants will receive oral and written information about the study and written formal consent will be collected.

The ongoing study for the comparison group: “Cycling with anabolic steroids: studying how large fluctuations in sex hormones affect brain chemistry, functional network, cognition and emotions” is approved by Norwegian Regional Committee for Medical Research Ethics (2013/601) and the Data Protection Officer at Oslo University Hospital (18/10139–3). Written consents are collected from all participants prior to inclusion. Participants are compensated with NOK 500 (≈$50) for taking part in the study and have the opportunity to discontinue the study at any point.

The research will be carried out according to the Helsinki declaration*.* Emphasis is placed on voluntary participation and that refraining from participation is possible at any stage in the study period prior to data publication without affecting the access to SUD outpatient treatment. All participants in the intervention are insured against adverse events attributable to study drug occurring during or after participation, through Project leader´s membership in the Norwegian Drug Liability Association. The participants in the intervention group are not compensated for taking part in the study, but study medication and physical health examinations in the study will not involve any costs. The participants will pay deductibles as patients in outpatient substance use disorder treatment and at potential follow-ups through somatic health services where indicated. All pathological findings of clinical relevance will be evaluated by physicians connected to the study and investigated further on indication.

## List of abbreviations

**Table:** 

AAS	Anabolic androgenic steroids
SUD	Substance Use Disorder
HPG	Hypothalamic-Pituitary-Gonadal
ASIH	Anabolic-androgenic Steroid Induced Hypogonadism
PCT	Post Cycle Therapy
PIEDs	Performance and Image Enhancing Drugs
CC	clomiphene citrate
SERM	Selective Estrogen Receptor Modulator
GnRH	Gonadotropin-Releasing Hormone
LH	Luteinizing Hormone
FSH	Follicle Stimulating Hormone
TRT	Testosterone Replacement Therapy
ALT	Alanine transaminase
AST	Aspartate transaminase
OUH	Oslo University Hospital
hCG	human Chorionic Gonadotropin
T	Testosterone
BMI	Body Mass Index
ECG	Electrocardiogram
BP	Blood Pressure
HR	Heart Rate
CAR	Carotid Artery Reactivity
DXA	Dual energy X-ray absorptiometry
TLM	Total Lean Mass
TFM	Total Fat Mass
VAT	Visceral Adipose Tissue
GLMM	General Linear Mixed Models
GAMM	General Alinear Mixed Models
RCT	Randomized Control Trial
CTCAE	Common Terminology Criteria for Adverse Events
SAE	Severe Adverse Events
SUSAR	Suspected Unexpected Severe Adverse Reaction

## Protocol validation

Not applicable

## Limitations

This non-randomized proof-of-concept intervention study with a small number of participants, is the first study to test off-label use of CC with the intention to restart endogenous testosterone production upon cessation of AAS use among men with AAS induced hypogonadism. The study will compare symptoms of AAS induced hypogonadism and other AAS-related side effects between the group receiving CC and a group of male AAS users not receiving the intervention. The comparison group differs from the intervention group as the participants may have less serious AAS use and less symptom burden during use as they mostly use AAS as cycles with an intention to cease use only temporarily. Moreover, the 12 months follow-up on examination of health risks in the intervention group may be too short as sperm quality is found to take up to 3 years to recover [15] and men with former AAS use is found to have lower plasma testosterone levels and more symptoms suggestive of hypogonadism than healthy controls several years after AAS cessation [30]. Finally, many health risks are associated with AAS use [[4], [5], [6]], and measures of a selection of health risks are included in this study. However, detailed, and frequent monitoring using objective biological measures is a strength and the study may provide valuable clinical insights, enabling the exploration of whether adjustments are needed for the intervention. Furthermore, the results may be used to determine the sample size and informing the design of future RCT or case comparison studies.

## Supplementary material *and/or* additional information [OPTIONAL]

None.

## CRediT authorship contribution statement

**Ingrid Amalia Havnes:** Conceptualization, Methodology, Investigation, Resources, Writing – original draft, Writing – review & editing, Visualization, Supervision, Project administration, Funding acquisition. **Hans Christian Bordado Henriksen:** Methodology, Investigation, Formal analysis, Data curation, Writing – original draft, Writing – review & editing, Visualization, Project administration. **Per Wiik Johansen:** Conceptualization, Methodology, Writing – review & editing. **Astrid Bjørnebekk:** Methodology, Investigation, Resources, Writing – review & editing, Supervision, Project administration, Funding acquisition. **Sudan Prasad Neupane:** Methodology, Supervision, Writing – review & editing. **Jonny Hisdal:** Methodology, Resources, Investigation, Writing – review & editing. **Ingebjørg Seljeflot:** Methodology, Resources, Investigation, Writing – review & editing. **Christine Wisløff:** Resources, Writing – review & editing. **Marie Lindvik Jørstad:** Writing – review & editing. **Jim McVeigh:** Methodology, Writing – review & editing. **Anders Palmstrøm Jørgensen:** Conceptualization, Methodology, Investigation, Resources, Writing – review & editing, Supervision.

## Declaration of Competing Interest

The authors declare that they have no known competing financial interests or personal relationships that could have appeared to influence the work reported in this paper.

## Data Availability

No data was used for the research described in the article.
